# Evolving Project ECHO: delivery of pediatric pain core competency learning for interprofessional healthcare providers

**DOI:** 10.3389/fpain.2023.1215811

**Published:** 2023-08-22

**Authors:** C. Lalloo, V. Mohabir, F. Campbell, N. Sun, S. Klein, J. Tyrrell, G. Mesaroli, J. Stinson

**Affiliations:** ^1^ Child Health Evaluative Sciences, Peter Gilgan Centre for Research and Learning, The Hospital for Sick Children, Toronto, ON, Canada; ^2^ Institute of Health Policy, Management and Evaluation, University of Toronto, Toronto, ON, Canada; ^3^ Department of Anesthesia and Pain Medicine, The Hospital for Sick Children, Toronto, ON, Canada; ^4^ Department of Anesthesiology and Pain Medicine, University of Toronto, Toronto, ON, Canada; ^5^ Department of Rehabilitation, The Hospital for Sick Children, Toronto, ON, Canada; ^6^ Department of Physical Therapy, University of Toronto, Toronto, ON, Canada; ^7^ Lawrence S. Bloomberg Faculty of Nursing, University of Toronto, Toronto, ON, Canada

**Keywords:** pediatric pain, Project ECHO, core competency, tele-education, distance education, continuing professional development, community of practice, interprofessional

## Abstract

**Introduction:**

Healthcare providers (HCPs) practicing in community settings are critical to improving access to pain care, yet there are significant gaps in training opportunities designed for interprofessional learners. Project Extension for Community Healthcare Outcomes (Project ECHO®) is an established model for delivering online HCP education through virtual clinics and cultivating a community of practice. However, to our knowledge, the integration of pain core competency education into the *ECHO®* model has not been previously attempted. This innovation could enhance the *ECHO®* model while also addressing the growing calls for more accessible interprofessional pain curricula. This paper describes efforts to implement and evaluate core competency curricula within the context of *Pediatric Project ECHO for Pain*, one of the first pediatric-pain focused ECHO programs in the world.

**Methods:**

Needs assessments informed curricula development. The first delivered core competency model consisted of synchronous webinar-style sessions while the second model included a mixture of asynchronous (eLearning course) and synchronous (virtual clinical debrief) elements. A convenience sample of HCPs was recruited from ECHO program registrants. Participants completed baseline and follow-up surveys to assess core competency acceptability as well as impact on knowledge and self-efficacy related to managing pediatric pain. Usability of the eLearning platform (model 2 only) was also evaluated. Surveys used 5-point Likert scales to capture outcomes. *A priori* targets included mean scores ≥4/5 for acceptability and ≥80% of learners reporting knowledge and self-efficacy improvements. The study received local research ethics approval.

**Results:**

The core competency was found to be highly acceptable to interprofessional learners (*n* = 31) across delivery models, surpassing *a priori* targets. Specifically, it was characterized as a worthwhile and satisfactory experience that was helpful in supporting learning. The core competency was also associated with improvements in knowledge and self-efficacy by 97% and 90% of learners, respectively. The eLearning platform was reported to have high usability with clinically realistic cases (100% of respondents) that were helpful to inform care delivery (94% of respondents).

**Conclusion:**

The integration of core competency learning within the *Project ECHO®* model was a successful approach to deliver pediatric pain education to interprofessional HCPs.

## 1. Introduction

Pain is a significant health problem for children and youth that can impair all aspects of life (1–4). Pediatric acute and chronic pain have differing profiles, with acute pain arising from tissue harm (e.g., surgery, injury, disease), which usually resolves as tissues heal. Timely pain management is essential to mitigate the risk of transition from acute to chronic pain (5, 6). Chronic pain is defined as pain lasting more than 3 months with significant emotional distress and/or functional disability (7–9). It is subdivided into chronic primary pain (i.e., disease in its own right, such as headache, abdominal pain, musculoskeletal pain) or chronic secondary pain (i.e., caused by another health condition such as juvenile idiopathic arthritis or sickle cell disease) (7–9). Chronic pain affects 1 in 5 children and youth, particularly from equity seeking populations (4, 7, 8, 10–12). Specialty tertiary care clinics generally manage children and youth severely impacted by pain. Unfortunately, prolonged wait times to access specialized pediatric pain management programs can have detrimental impacts on patients and families (13, 14).

In 2019, Canadian healthcare providers (HCPs) and families (children, youth, and caregivers) impacted by pain identified “better access to pain care” and “increasing healthcare provider training, knowledge, recognition, beliefs, attitudes, and communication” related to pediatric pain as top priorities (15). This need has been further articulated by policymakers and key stakeholders such as in the federal government's “Action Plan for Pain in Canada”, which has emphasized the critical need to engage HCPs from primary and secondary care settings in managing pediatric acute and chronic pain (12). However, there are significant gaps in available pain education for HCPs and a need for more opportunities to support interprofessional training in pain management across Canada and worldwide (10, 16, 17).

Since the 1990's, the International Association for the Study of Pain and the global pain community have recognized the importance of core pain curricula for interprofessional HCPs (18–21). Specific to pediatrics, a 2019 review found that, “education regarding the assessment and treatment of pain in children is needed across all relevant disciplines including within medicine, nursing, physiotherapy, and psychology” (p. 4) (17). This review also identified that “…innovative pain education programmes are generally not well implemented; both accessibility to and assessment of these programmes must be improved to facilitate positive changes in current practice” (p. 4) (17).

*Project ECHO® (Extension for Community Healthcare Outcomes)* is an established model for delivering online HCP education through virtual clinics and cultivating a supportive community of practice (22, 23). *ECHO®* uses a “Hub-and-Spoke” structure, wherein the Hub (i.e., a specialty interprofessional team) regularly connects via videoconference with multiple Spokes (i.e., community-based HCPs) to learn together with the shared goal of enhancing local patient care. The traditional *ECHO®* model is centred on virtually delivered “TeleECHO clinics” wherein a brief didactic presentation is followed by de-identified case presentation from a community HCP and facilitated group discussion to generate best practice recommendations for case management. The presenting HCP then has autonomy to apply the recommendations to their specific patient case, while other Spoke learners can reflect on how the discussed principles can be applied to their own practices. The overarching goal of *ECHO®* is to empower HCPs with training, mentorship, and support to locally manage their patients with specialized health needs within the framework of a virtual community of practice. ECHO programs have been developed to support a wide variety of health conditions, including acute and chronic pain (22, 24, 25).

*Pediatric ECHO® For Pain*, based in Ontario Canada, is one of the largest pediatric pain-focused *ECHO®* programs in the world (26). Program scope is inclusive of multimodal, evidence-based approaches to support interdisciplinary management of pediatric acute, chronic, and transitional pain. Since 2017, this program has delivered more than 100 TeleECHO clinics to an interprofessional audience of over 1,800 HCPs from 27 different disciplines. These TeleECHO clinics are associated with significant improvements in interprofessional HCP pain knowledge and self-efficacy as well as positive practice impacts (26).

To our knowledge, the integration of pain core competency education (i.e., focus on foundational education) into the *ECHO®* model has not been previously attempted. However, *Pediatric ECHO® For Pain*, with its robust infrastructure and interprofessional audience, offers a timely opportunity to explore the implementation of core competency alongside the prototypical TeleECHO clinics. This innovation could enhance the *ECHO®* model while also addressing the growing calls for more accessible interprofessional pain curricula.

Our group has previously reported on pilot delivery of a pain core competency within *Pediatric ECHO® For Pain* (26). While this model was positively received by attendees, many HCPs found it challenging to find the time to participate in the core competency in addition to the TeleECHO clinics. For instance, in a survey probing on reasons for low program attendance, over 50% of respondents (*n* = 123) cited lack of availability during the scheduled sessions (27).

In response to these identified learner needs, the program has trialed different core competency delivery models, including both asynchronous and synchronous elements. In this paper, we will describe efforts by *Pediatric ECHO® For Pain* to refine the delivery of core competency curricula for interprofessional HCPs related to managing pain in children and youth. Evaluation data from varying delivery models will be presented related to acceptability as well as impacts on knowledge and self-efficacy. These data will inform recommendations for integrating core competency learning within the *Project ECHO®* model as well as broader implications for the HCP pain education landscape.

## 2. Methods

This study received research ethics board approval from The Hospital for Sick Children (#1000057321) and adhered to the Canadian Tri-Council Policy Statement on Ethical Conduct for Research Involving Humans.

### 2.1. Description of delivery models

*Model 1 (Synchronous):* The initial offering of Core Competency consisted of webinar-style sessions conducted live over Zoom. Interprofessional HCPs could register for the sessions and join remotely from their personal web-enabled device (e.g., computer, tablet, smartphone). Each session was facilitated by a member of the Pain Hub team, located at The Hospital for Sick Children (SickKids), which is the largest pediatric tertiary care hospital in Canada. Sessions were 60 min in duration and the curriculum was delivered over 8 installments delivered once a week. The curriculum content was informed by a previously reported online needs assessment (26).

*Model 2 (Hybrid of Asynchronous and Synchronous):* The subsequent Core Competency offering consisted of a mixture of asynchronous and synchronous elements. An asynchronous eLearning course dedicated to the fundamentals of pediatric pain management was created in partnership with AboutKidsHealth (i.e., the patient and family health education group within SickKids). Curriculum content was informed by a needs assessment of interprofessional learners registered for the *Pediatric ECHO® For Pain* program (26). The eLearning course consisted of four individual modules designed to offer an interactive user experience through embedded resources, quizzes (e.g., multiple choice, multiple responses, fill-in-the-blank, matching), and case studies. The eLearning platform (Articulate Rise 360) had a responsive design and was accessible on any web-enabled device (e.g., desktop, tablet, mobile). The synchronous model component was a 60-minute clinical debrief of the Core Competency eLearning content, conducted live over Zoom. The debrief was offered to HCP learners as an opportunity to discuss the curriculum content with peer learners and the Pain Hub team.

### 2.2. Model implementation and evaluation

Each Core Competency model was implemented as part of the program-level offerings of *Pediatric ECHO® For Pain*. The synchronous model was delivered between November 2017 and January 2019, while the hybrid model was delivered between October and December 2021. Differences in duration of model delivery were a function of requirements from the program funder (Ontario Ministry of Health).

HCP learners who registered for either model completed a baseline survey to assess their expectations as well as current knowledge and self-efficacy related to managing pediatric pain. Learners who either attended at least one synchronous session (model 1) or completed at least one eLearning module (model 2) were sent a follow-up survey to assess acceptability as well as any changes in knowledge or self-efficacy since starting the program. All survey administration was managed using REDCap, a secure electronic data collection tool hosted at SickKids (28).

### 2.3. Data analysis

Quantitative survey data were summarized using descriptive statistics. Where item-level survey response options differed between models (e.g., 7-point Likert vs. 5-point Likert), a merged scale was used. For instance, the response items of “2 = disagree” and “3 = somewhat disagree”, drawn from a 7-point Likert agreement scale, were re-coded as “2 = disagree” within a 5-point Likert scale. The *a priori* targets for assessed constructs were mean acceptability scores ≥4 (possible scores ranged from 1 to 5); ≥80% of learners reporting improvements in knowledge related to managing pediatric pain; and ≥80% of learners reporting improvements in confidence related to managing pediatric pain. In addition to the constructs described above, the usability of the eLearning platform (Model 2 only) was also assessed. The *a priori* targets for platform usability were mean score ≥4 for ease of use (possible scores ranged from 1 to 5); ≤5% of learners reporting major technical issues; and ≥80% of learners describing the exemplar patient cases within the modules as both clinically realistic and helpful in informing their delivery of care. Data were exported from REDCap and analysis was conducted using Microsoft Excel Version 16.60 by authors CL and VM.

## 3. Results

### 3.1. Characteristics of HCP learners

Demographic characteristics of the HCP learners are summarized in Table 1. Many expectations for the Core Competency learner experience were shared across models (*n* = 31), including:

• Expand knowledge and confidence with up-to-date information to guide clinical practice (100%)
• Integration of case-based learning (88%)
• Joining an interactive community of practice that accommodates different learning styles (69%)

**Table 1:** Demographic characteristics of core competency learners (*N* = 31).

Characteristic (*n*, %)	Model 1 (Synchronous), *n* = 15	Model 2 (Hybrid), *n* = 16
Profession		
Child Life Specialist	0 (0)	4 (25)
Nurse Practitioner	2 (13)	1 (6)
Registered Nurse	3 (20)	9 (56)
Rehabilitation Specialist		
(e.g. physiotherapist)	6 (40)	1 (6)
Physician	3 (20)	1 (6)
Missing	1 (7)	0 (0)
Gender Identity		
Man	0	0 (0)
Prefer not to answer	0	1 (6)
Woman	15 (100)	15 (94)
Race		
Black	1 (7)	1 (6)
East Asian	0 (0)	1 (6)
Indigenous	0 (0)	1 (6)
Prefer not to answer	0 (0)	2 (13)
South Asian	3 (20)	0 (0)
White	11 (73)	11 (69)
Age		
0–19 years	0 (0)	1 (6)
20–29 years	3 (20)	4 (25)
30–39 years	6 (40)	4 (25)
40–49 years	4 (27)	3 (19)
50–59 years	2 (13)	3 (19)
Prefer not to answer	0 (0)	1 (6)
Years of Practice		
Less than 1 year	2 (13)	3 (19)
1–4 years	3 (20)	2 (13)
5–10 years	1 (7)	5 (31)
Greater than 10 years	7 (47)	5 (31)
Prefer not to answer	2 (14)	1 (6)
Primary Practice Setting		
Academic Hospital	9 (60)	5 (31)
Community	1 (7)	4 (25)
Family Health Team	1 (7)	1 (6)
Non-Academic Hospital	1 (7)	2 (13)
Other	0 (0)	2 (13)*
Private Practice	3 (20)	1 (6)
Prefer not to answer	0	1 (6)

* Other: Hospice; Not specified.

### 3.2. Acceptability

All learners (*n* = 31) characterized the Core Competency as a worthwhile and satisfactory experience. Average Likert scores for this construct, which could range from 1 (“strongly disagree”) to 5 (“strongly agree”), were 4.4 ± 0.5 for Model 1 and 4.7 ± 0.5 for Model 2, respectively.

The Core Competency was also characterized as effective and helpful in supporting learning by nearly all participants (*n* = 30; 97%). Average Likert agreement scores for this construct were 4.3 ± 0.6 for Model 1 and 4.6 ± 0.5 for Model 2, respectively.

Nearly all Model 1 participants (*n* = 13/14; 93%) agreed that the Core Competency training environment created a supportive community of practice. Given the more independent learning style of Model 2 (i.e., asynchronous eLearning with option for live group debrief), those participants were asked to characterize their perceptions about level of peer interaction. Of the *n* = 8 respondents to this item, 7 (88%) felt that Model 2 included “the right amount of opportunities for peer-to-peer learning”.

### 3.3. Knowledge and self-efficacy impacts

**Figure 1:**
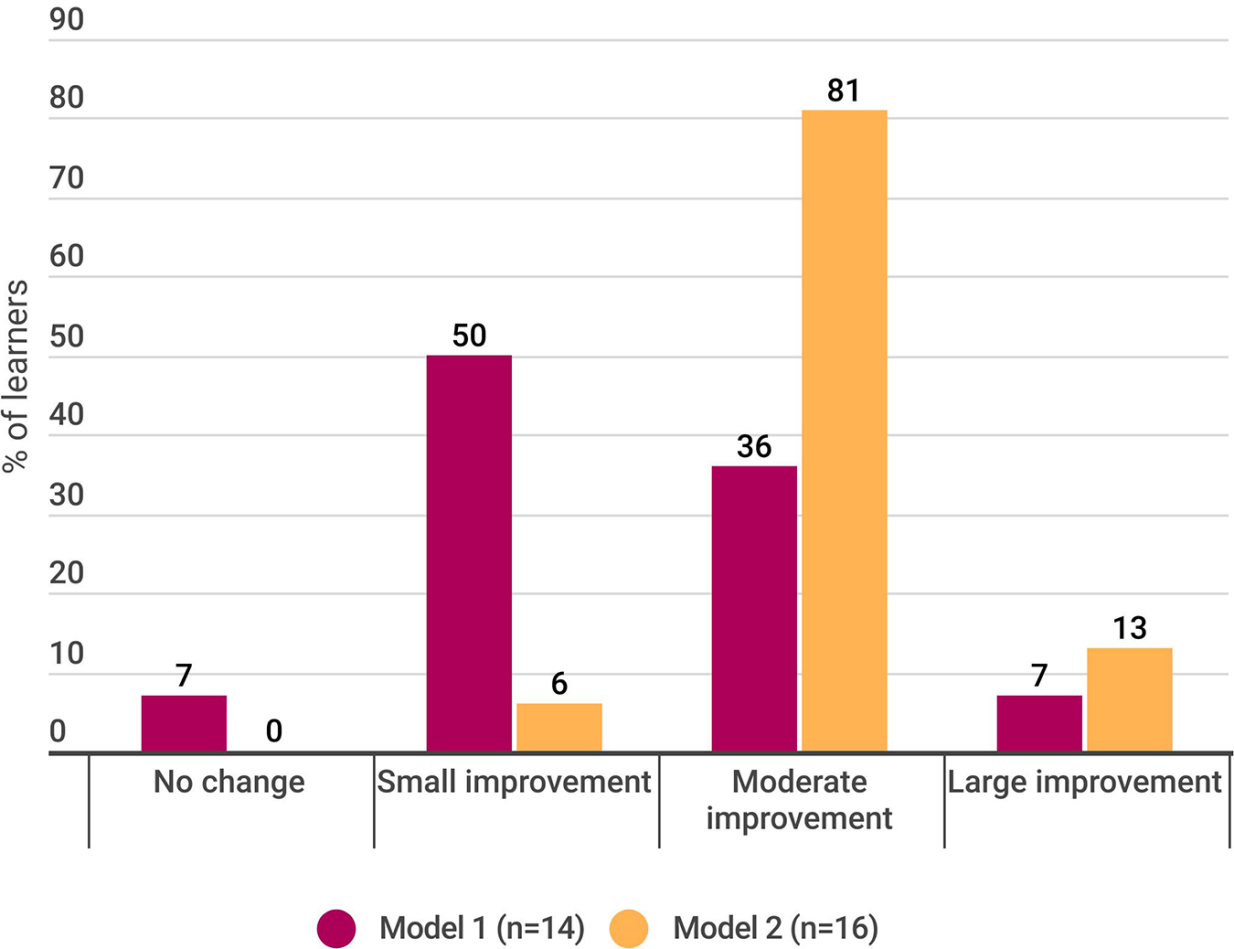
Impact of core competency on learner knowledge of pediatric pain.

Nearly all learners (29/30; 97%) reported improvements in their knowledge related to managing pediatric pain. The relative magnitude of knowledge impact across models is illustrated in Figure 1.

**Figure 2:**
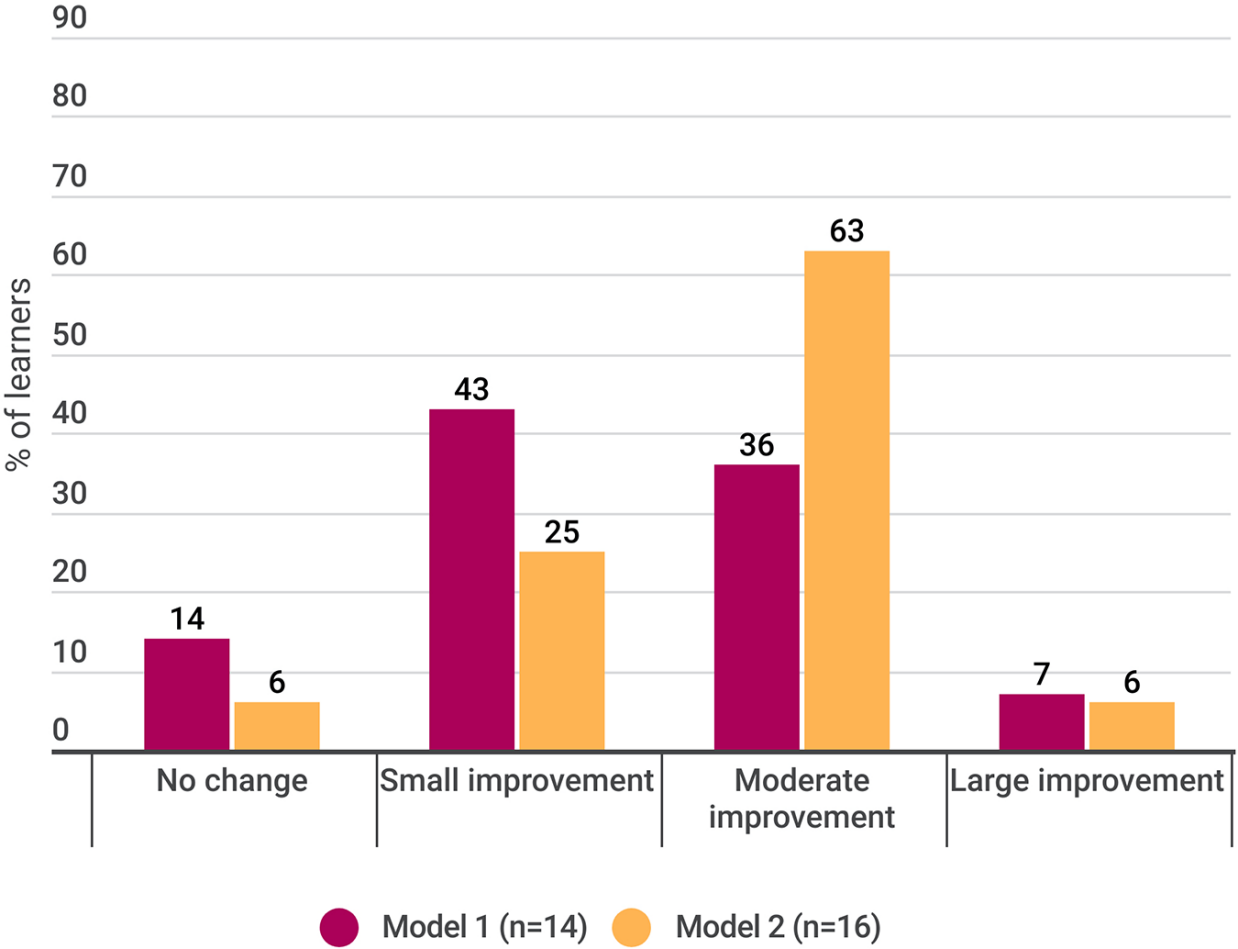
Impact of core competency on learner confidence in managing pediatric pain.

Similarly, most learners (27/30; 90%) reported improvements in their self-efficacy or confidence in clinical management of children and youth with pain. The relative magnitude of self-efficacy impact across models is illustrated in Figure 2.

### 3.4. Usability of eLearning platform and anticipated applications

All Model 2 learners (*n* = 16) characterized the eLearning platform as easy to use. The average Likert score for this item, which could range from 1 (“very difficult”) to 5 (“very easy”) was 4.5 ± 0.7. No major technical issues were encountered with the eLearning platform during the delivery period.

The exemplar pediatric pain cases within the eLearning modules were well-received with all Model 2 participants (*n* = 16) describing them as clinically realistic. Similarly, nearly all participants (*n* = 15; 94%) characterized the patient cases within the modules as helpful to inform their patient care.

Planned applications for the eLearning modules by learners (*n* = 16) included:

• Gaining new knowledge and skills, such as reviewing an eLearning course for their own interest and/or to enhance their clinical practice (94%);
• As a refresher to stay up-to-date on best practice guidelines (88%);
• As a recommended resource for colleagues (77%);
• As a resource for local trainees (41%); and
• Just in time training such as reviewing a specific module prior to seeing a complicated pain case (24%).

## 4. Discussion

This study sought to evaluate the delivery of core competency curricula for interprofessional HCPs related to managing pain in children and youth. *Project ECHO®*, an established model for delivering accessible virtual education, was adapted to integrate different core competency modalities. The first model consisted of synchronously delivered sessions while the second model used a hybrid approach of asynchronous eLearning modules paired with a synchronous debrief.

The *Pediatric ECHO® For Pain* core competency was found to be highly acceptable to interprofessional learners (*n* = 31) across delivery models, surpassing *a priori* targets. Specifically, the pain core competency was characterized as a worthwhile and satisfactory experience that was helpful in supporting learning. The core competency was also associated with reported improvements in knowledge and self-efficacy by 97% and 90% of learners, respectively. The eLearning platform utilized for Model 2 was reported to have high usability, surpassing *a priori* targets. Moreover, the demonstrative patient cases within the eLearning platform were characterized as clinically realistic (100% of respondents) and helpful to inform care delivery (94% of respondents).

Overall, the integration of core competency learning within the *Project ECHO®* model was a successful approach to deliver pediatric pain education. While the traditional *ECHO®* model concentrates on the TeleECHO clinic as a learning modality (i.e., didactic paired with patient case discussion), our data suggest that the model can be leveraged to also offer foundational education to an interprofessional learning community. Nascent and established *ECHO®* programs may wish to consider the integration of core competency elements into their curricula. A stepwise approach including needs assessment to understand learner requirements, environmental scan of existing educational opportunities, and pilot evaluation is recommended. Our group also recommends the inclusion of asynchronous components such as eLearning modules to enhance accessibility (e.g., opportunity to reinforce knowledge uptake through on-demand access to content, accommodation of different learning styles, optimizing screen readability, option for alternative text).

Strengths and Limitations: Study participants represented numerous professions and clinical disciplines, reflecting the real-world care management of pediatric pain, and enhancing the generalizability of findings to a broad group of HCP learners. Another study strength was the evaluation of different delivery modalities for pain core competency within the ECHO model. A limitation of this study was low diversity in terms of gender identity and race of participants. Given this was a pilot study using a convenience sample of learners, future evaluations will seek to purposively include a larger and more heterogeneous group of HCP learners. Findings are also subject to the limitations of self-reported knowledge and self-efficacy scores due to the lack of validated tools designed to measure these constructs across the varied healthcare professions that care for children with pain.

A 2023 survey study sought to characterize the continuing professional development needs of Canadian HCPs related to pain management among an interprofessional sample of *n* = 230 HCPs, including nurses, pharmacists, physicians, rehabilitation therapists, and dentists from a variety of practice settings (20). In this study, the most frequent pain education activities were reading journal articles (56%), online independent learning (44%), and attending hospital rounds (43%). Overall, 17% of respondents did not complete any pain learning activities in the past 12 months. Participants also stressed the need for more resources related to the care of children and youth with different pain conditions. The authors concluded that, “Canadian post-licensure [HCPs] require greater access to and participation in interactive and multimodal methods of continuing professional development to facilitate competency in evidence-based pain management” (p. 1). There is an opportunity for the *Pediatric ECHO® For Pain* core competency to begin to address this need related to pediatric pain. The eLearning modules, focused on headache, chronic widespread pain, functional gastrointestinal pain, and needle poke pain respectively, are now publicly available at https://sickkids.echoontario.ca/elearning/. Synchronous core competency sessions are continually offered through the program at no-cost to learners (see: https://sickkids.echoontario.ca for curriculum details).

Recently, Agley and colleagues completed a comprehensive qualitative study of five different ECHO programs with the aim of better understanding the model and identifying areas for improvement in implementation (29). A key recommendation was to “consider and experiment with ways that barriers to access can be overcome without diluting the model” (p. 7) with suggested solutions such as recording didactics for more convenient access. The pain core competency begins to address this identified need by offering on-demand access to session recordings (model 1) as well as eLearning modules (model 2). Future uptake of the resources amongst the ECHO community will be assessed through ongoing aggregate-level analytics to inform further core competency improvements.

The guiding principles of the Project ECHO model include amplification (i.e., using technology to leverage scarce resources), promotion of best practices (i.e., to reduce disparities in care), case-based learning, and continuous data collection to monitor program impacts (22). The core competency is aligned with each of these principles and may enhance the model by offering a new multimodal pathway to disseminate knowledge, which can then be applied locally to patient care. The ECHO model is also hypothesized to support “force multiplication” wherein learners eventually become local topic experts who can informally mentor colleagues in their community (22). The extent to which the pain core competency is associated with these more distal outcomes will be explored through future research.

Future research should also focus on the relationship between core competency curricula for pediatric pain and HCP practice implementation. Although the core competency model has evidence of positive impacts on HCP knowledge and self-efficacy, downstream effects on the direct care of patients and families are currently unknown. Future research will also examine the relationship between participation in the ECHO core competency education and subsequent engagement with TeleECHO clinics.

## 5. Conclusion

*Pediatric Project ECHO^®^ For Pain* has innovated the ECHO model by integrating pain core competency. Adaptations of the competency model have sought to refine the delivery of accessible, convenient, and useful pediatric pain education. This advancement has demonstrated value for interprofessional HCPs who manage children and youth with pain needs.

## Data Availability

The raw data supporting the conclusions of this article will be made available by the authors, without undue reservation.
